# Plasma-treated PLA incorporated with purple tomato anthocyanins-based coating to fabricate bilayer indicator films for monitoring the freshness of minced pork

**DOI:** 10.1016/j.fochx.2026.104247

**Published:** 2026-07-29

**Authors:** Guorui Zhou, Yana Li, Beihai Wang

**Affiliations:** School of Mechanical Engineering, Wuhan Polytechnic University, Wuhan 430023, China

**Keywords:** Polylactic acid, Dual-layer, Purple tomato anthocyanin, Smart packaging

## Abstract

In this study, plasma-treated polylactic acid (PLA) was employed as the substrate, and a mixture of purple tomato anthocyanins (PTA) and polyvinyl alcohol (PVA) was utilized as a functional coating to fabricate a novel, environmentally friendly, multifunctional bilayer smart indicator film. The results indicate that, with increasing PTA concentration, the elongation at break varies from 94.98% to 159.16%; water vapor permeability decreases from 9.3 × 10^−11^ to 7.97 × 10^−11^ g·m/(m^2^·s·Pa); oxygen permeability declines from 1.18 to 0.65 cm^3^/(m^2^·d·0.1 MPa); total migration increases from 0.62 to 2.31 mg/dm^2^; the water contact angle rises from 57.57° to 69.76°; and the pH-sensitive ΔE increases from 4.46 to 16.70. During pork mince packaging, the color of films (PTA content ≥10 g/dL) shifted from light purple to light green as the meat deteriorated, highlighting its smart indicator function. The developed film enhances PLA's barrier properties, integrates smart indicator characteristics and expands PLA's application prospects in food packaging.

## Highlights

• Plasma-treated PLA with PTA/PVA coating resulting to bilayer-structure films.
• Enhanced PLA barrier properties by PTA/PVA coating.
• Monitoring the minced pork freshness well via visual color change.
• Optimal PTA content of 10 g/dL for smart packaging application.
• Significantly outperforms pure PLA or PVA films in comprehensive performance.

## Graphical abstract

**Figure:**
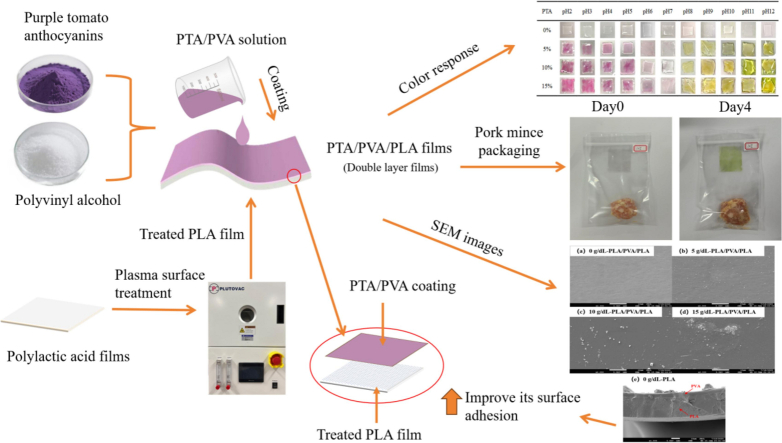

## 1 Introduction

Smart packaging has garnered significant attention for its ability to enable real-time quality assessment through visual indicators, including colorimetric responses to freshness parameters, spoilage processes, or internal gas environments (e.g., NH₃, CO₂) (Han et al., 2023). Environmentally friendly polymers are typically chosen as carriers and combined with pH-sensitive dyes to prepare smart indicator labels. Common carriers include chitosan, polyvinyl alcohol (PVA), starch, sodium alginate, cellulose, and others. However, these single-film materials suffer from poor mechanical properties, weak barrier performance, susceptibility to swelling, poor water resistance, and limited functionality. Consequently, the development of novel composite materials has become a key research focus (Özbaş & Özgen, 2026; Abedi-Firoozjah et al., 2025; Basdeki et al., 2025; Kamthai and Magaraphan, 2019; Paunonen, 2013; Yutong & Yana, 2024).

Polylactic acid(*PLA*)is a highly promising biodegradable material. Compared with common natural polymers, its excellent mechanical properties are often used as the base material, combined with functional coatings to prepare multi-layer composite materials for food packaging (Paunonen, 2013). Chen, et al. (Yutong & Yana, 2024), have developed a high-barrier antibacterial film for active packaging based on multi-layer films composed of PLA/PBAT and sodium alginate. Zhao et al. (Zhao et al., 2023) studied a double-layer antibacterial film composed of an inner layer of chitosan (PM)/polyvinyl alcohol (PVA) and an outer layer of polylactic acid (PLA). Pirsa et al. (Pirsa & Asadi, 2021) prepared a multilayer indicator film composed of polylactic acid (PLA), titanium dioxide (TiO_2_), and lycopene (PLA/TiO2/Lyc). The incompatibility between PLA's hydrophobicity and the hydrophilic coating remains unresolved.

Anthocyanins, a type of water-soluble flavonoid pigment widely present in plants, and polyvinyl alcohol, due to their good film-forming properties, water-solubility, and food safety, are widely used in the food packaging field. Kang et al. (Kang et al., 2018)developed a new colorimetric membrane for monitoring the freshness of shrimp by coating mulberry anthocyanin (MBA) on the inner side of a multi-layer film made of ethylene-vinyl alcohol copolymer-montmorillonite (EVOH-MMT).Li et al. (Li et al., 2024) prepared a double-layer smart detection membrane based on polyvinyl alcohol-chitosan/nano-ZnO/sodium alginate (SA) combined with anthocyanin extract (anthocyanin chloride). Wan et al. (Wan Zullkiplee et al., 2025) produced biodegradable films using pH-sensitive anthocyanin extracts from *Passiflora suberosa*. Being different with the poor stability of common anthocyanins (degradation under light, heat, and pH), purple tomato anthocyanin (PTA) contains a large amount of acylated anthocyanins. This structure can enhance molecular stability and reduce degradation caused by light exposure, high temperature, and pH value changes (Colanero et al., 2020). Although anthocyanins have been used in smart films, there are currently no reports on the use of purple tomato anthocyanin (PTA) coatings based on PLA for smart packaging.

Purple tomato anthocyanins (PTA) are structurally distinct from anthocyanins derived from other common sources, such as purple potato, mulberry, and roselle. The anthocyanins in purple tomato are predominantly acylated, with glycosylation and acylation being the primary structural modifications (Wang et al., 2020). Specifically, petunidin-3-(trans-p-coumaroyl)-rutinoside-5-glucoside and malvidin-3-(trans-p-coumaroyl)-rutinoside-5-glucoside have been identified as the main anthocyanin components in purple tomato cultivars such as Indigo Rose. In contrast, purple potatoes are also rich in acylated anthocyanins, with petanin (petunidin-3-O-[6-O-(4-O-*E*-p-coumaroyl-O-α-L-rhamnopyranosyl)-β-D-glucopyranoside]-5-O-β-D-glucopyranoside) as a commonly reported representative, whereas mulberries contain predominantly non-acylated anthocyanins (Jokioja et al., 2021). Roselle anthocyanins, on the other hand, have been evaluated as pH indicators in intelligent film systems, exhibiting color transition patterns from red to reddish-pink (pH 3–4), pink (pH 5–6), purple (pH 7–10), and green-yellow (pH 12) (Nguyen et al., 2024). The acylation of anthocyanins is known to enhance color stability, alter absorption and bioavailability, and improve resistance to degradation compared to non-acylated counterparts. Therefore, the unique acylated structure of PTA may confer distinct physicochemical properties and pH-responsive behavior when incorporated into polymer film matrices, distinguishing it from other anthocyanin sources commonly used in intelligent packaging applications.

However, the inherent hydrophobicity and low surface energy of PLA hinder its bonding with hydrophilic functional coatings. This interfacial incompatibility can lead to delamination and peeling in the final composite film, thereby compromising its performance. To address this issue, plasma surface treatment has emerged as an efficient, dry, and environmentally friendly method for polymer surface modification (Sanchis et al., 2006; Yana & Guorui, 2025). The plasma treatment process on the surface of PLA is as follows: (1) The plasma is excited; (2) The surface of the PLA film is bombarded with reactive particles; (3) The surface modification is achieved. Through reactions with reactive species in the plasma, plasma treatment introduces polar functional groups onto the PLA surface, thereby enhancing its surface hydrophilicity and adhesion (Yang et al., 2024).

Therefore, this study aims to investigate whether combining a plasma-treated polylactic acid (PLA) substrate with a coated bilayer film of purple tomato anthocyanins (PTA) and polyvinyl alcohol can simultaneously address the single function for monolayer indicator and interfacial issues in intelligent packaging, and to characterize its performance in real-time freshness monitoring of pork (Bahramian et al., 2025; Javdani et al., 2025).

## 2 Materials and methods

### 2.1 Materials

PTA was from (Zhejiang Bison Biotechnology, Ltd., Jiaxing, China). These sub-stances were extracted from purple tomato fruits using an efficient purification method, resulting in a purity of 54 g/dL. Polyvinyl alcohol (PVA, 99 g/dL alcoholysis degree) was sourced from (Shanpu Chemical Co., Ltd., Shanghai, China) (CAS: 9002-89-5). PLA was purchased from (Hengqin Huizefeng Packaging Materials Co., Ltd., Zhuhai, China) (CAS: 26100–51-6). Glacial acetic acid (analytical purity) was purchased from (Bohuatong Chemical Products Sales Center, Tianjin, China) (CAS: 64–19-7). DPPH (1,1-diphenyl-2-trinitrophenylhydrazine) containing 95 g/dL free radicals was sourced from (Sinopharm Chemical Reagent Co., Ltd., Shanghai, China) (CAS: 1898-66-4). The pork mince was purchased from the Zhongbai Warehouse in Wuhan, China. All chemicals used were high-purity analytical grade chemicals. The solvent in all formulations was deionized water.

### 2.2 Preparation of PTA/PVA/PLA composite film

PTA/PVA/PLA composite films were prepared by a coating method. First, 20 g of PVA was added to 200 mL of deionized water and stirred in a water bath at 90 °C for 5 h at 800 rpm. Then, 0 g, 1 g, 2 g, and 3 g of PTA were added to the PVA solution and stirred at 800 rpm for 5 h at room temperature. Subsequently, the solutions were placed in a thermostatic shaker for 1 h to remove air bubbles to obtain 0 g/dL-PTA/PVA, 5 g/dL-PTA/PVA, 10 g/dL-PTA/PVA, and 15 g/dL-PTA/PVA solutions. The PLA films were cut into 15 cm × 15 cm squares and treated in a plasma processing equipment (PLUTO-M, Shanghai Peiyuan Co., Ltd.) to obtain plasma-pretreated PLA films, in which, a 5-min processing time and the power of 120 W were employed due to resulting in the optimal wettability for PLA surface based on our previous experimental results (Yana & Guorui, 2025). Finally, 2 mL of the PTA/PVA mixture was dropped onto the plasma-treated PLA films using a dropper and spread uniformly with a coating rod. The films were dried in an oven at 40 °C for 8 h to obtain 0 g/dL-PTA/PVA/PLA, 5 g/dL-PTA/PVA/PLA, 10 g/dL-PTA/PVA/PLA, and 15 g/dL-PTA/PVA/PLA bilayer composite films with a thickness of 50 ± 1 μm.

### 2.3 Characterization of PTA/PVA/PLA composite films

#### 2.3.1 Mechanical properties and barrier properties

According to the method specified in ASTM D882–18, the mechanical properties of the film were measured at 25 °C using an electric universal testing machine (UTM4104, SUNS, Shenzhen, China). The film samples, with dimensions of 10 mm × 15 mm, were tested at a stretching speed of 50 mm/min. Each sample was tested for tensile strength (TS) and elongation at break (EAB), with a total of 5 samples tested. The mean values were then taken as the final test data (Yana et al., 2024).

The water vapor permeability (WVP) and oxygen permeability (OP) of the film were determined using a moisture permeameter (W3/031×, Labthink, Jinan, China) and an oxygen permeability meter (N530L, GBPI company, China), respectively. Each sample was simultaneously subjected to three parallel tests (Liu et al., 2018).

#### 2.3.2 Water contact angle, Fourier transform infrared spectroscopy, morphology and thermal stability

The water contact angle (WCA) of the film was determined by a contact angle tester (JCY-3, Shanghai Fangrui Instrument Co., LTD., Shanghai, China). According to the method provided by Ren et al. (Ren et al., 2022), the test water drop was 2 μl, and the WCA was recorded 5 s later. The experiment was repeated 3 times, and the average value was taken.

Fourier transform infrared (FTIR) spectra of the films were measured using a Fourier transform infrared spectrometer (WQF-530 A, Beifen Ruili Analytical Instrument Co., Ltd., Beijing, China) in the range of 4400–400 cm^−1^with a resolution of 4 cm^−1^ (Liu et al., 2023).

Surface morphology of the film was observed using a scanning electron microscope (SEM, SU-8010, HITACHI Co., Ltd., Japan). The film was mounted on a specimen holder, subjected to gold sputtering in the ion sputtering layer for 2 min, and then photographed (Wang, Lu, et al., 2024).

Thermal stability of the film was evaluated through thermogravimetric analysis (TGA) using a simultaneous thermal analyzer (HQT-4PLUS, Beijing Hengjiu Experimental Equipment Co., Ltd., Beijing, China) in alumina crucibles. Samples (10 ± 0.5 mg) were scanned from 35 °C to 600 °C at a constant heating rate of 10 °C/min under a nitrogen environment (50 mL/min) (Forghani et al., 2023).

#### 2.3.3 Antioxidant properties and release behavior of PTA

The antioxidant capacity of the films was assessed using the DPPH(1,1-diphenyl-2-picrylhydrazyl) assay. The film (50 mg) was added to 10 mL of 95 g/dL ethanol solution, and was extracted for 3 h in an ultrasonic cleaner at 50 °C. Then, 0.1 mL of the extract was mixed with 5 mL of DPPH solution (100 ppm). A mixture of 0.1 mL of anhydrous ethanol solution with 5 mL of DPPH solution (100 ppm) was prepared as the blank group. The reactions were conducted in the dark at room temperature (25 ± 2 °C) for 30 min (Schmitz & Moura, 2025). The absorbance of the extracts was measured at 517 nm, with three replicates for each sample. The DPPH radical scavenging activity (DRSA) was computed using the formula (1).

(1) $$\mathrm{DRSA}\left(\%\right)=\frac{A_{0}-A_{S}}{A_{0}}\times 100\%$$

In the formula: AS represented the absorbance of the experimental group; A0 represented the absorbance of the blank group.

The film (4 cm × 4 cm) was placed in a conical flask containing 100 mL of ethanol-water solution (95 g/dL, as a simulated food simulant). The flask was then placed in an incubator shaker (125 rpm, 25 °C) under dark conditions (Schmitz & Moura, 2025). Samples were periodically taken from each solution, and the absorbance at 292 nm was measured using a UV–Vis spectrophotometer, after which the solution was immediately returned to the conical flask. The amount of PTA released at the corresponding time was calculated based on the standard curve, and the cumulative release rate of the film was calculated according to formula (2).

(2) $$\text{Accumulative release}\left(\%\right)=\frac{M_{t}}{M_{0}}\times 100\%$$

M_t_ and M_0_ denote the amount of PTA released at time t and the total amount of PTA in the entire composite film, respectively.

#### 2.3.4 pH-response, sensitivity to ammonia and acetic acid, and color reversibility

The prepared PTA/PVA/PLA films (3 cm × 3 cm) were exposed to buffer solutions of pH 2–12. The films were immersed in the pH solution for 60 s and then photographed. At the same time, the L*, a*, and b* of the films as a function of pH was determined using a colorimeter (CR-10, Konica Minolta, Japan) (Wang, Zhang, et al., 2024). The value of the total color difference (∆E) was calculated according to Eq. (3).

(3) $$\mathrm{\Delta E}=\sqrt{{\left(L^{*}-L_{0}\right)}^{2}+{\left(a^{*}-a_{0}\;\right)}^{2}+{\left(b^{*}-b_{0}\;\right)}^{2}\;}$$

where L*, a*, and b* are the values of the samples; L_0_, a_0_, and b_0_ represent the original values of the standard white plate, and the values of L0, a0, and b0 were 92.9, 0.32, and 0.33, respectively.

First, 80 mL of ammonia solution and 80 mL of acetic acid solution were added to two separate conical flasks. Each sample was then placed 1 cm above the solution. Three parallel samples were prepared, and a camera was used to record the color changes of the films over 5 min. Finally, the R, G, and B values for each sample were recorded using the Pixie program on a Windows system. This procedure was repeated three times, with photographs taken to observe the color changes of the films (Lu et al., 2024). Subsequently, the sensitivity of the films to ammonia and acetic acid (SRGB) was calculated using formula (4).

(4) $$S_{\mathrm{RGB}}=\frac{\left(R_{a}-R_{b}\right)+\left(G_{a}-G_{b}\right)+\left(B_{a}-B_{b}\right)}{R_{a}+G_{a}+B_{a}}\times 100$$

where Ra, Ga, and Ba denote the initial parameters of the film, while Rb, Gb, and Bb represent the parameters obtained after exposure to ammonia solution and acetic acid solution environments.

Eighty milliliters of concentrated ammonia solution and eighty milliliters of glacial acetic acid solution were placed in two separate conical flasks (Ezati et al., 2020). First, the film was suspended in the flask containing the ammonia solution and kept at 25 °C for 3 min to expose it to ammonia vapor. Subsequently, the film was suspended in the glacial acetic acid solution and kept at 25 °C for 3 min. This cycle was repeated three times to observe the color changes of the film (Ma et al., 2017).

#### 2.3.5 Overall migration tests and biodegradation tests

In accordance with EU No 10/2011, the films were cut into 3 × 3 cm pieces and placed in 200 mL glass beakers, which were sealed with plastic wrap (polyvinylidene chloride). Each beaker was filled with 100 mL of the following food simulants: deionized water to represent aqueous foods, 10% ethanol to represent low-alcohol foods, and 4% acetic acid to represent acidic foods (EFSA Panel on Food Additives and Nutrient Sources added to Food (ANS), 2013; Redfearn & Goddard, 2023). After incubation at 40 °C for 10 days, 100 mL of the simulant was transferred using an overall migration and non-volatile residue tester (AUTO ZF3600, GBPI, Guangzhou, China) to an evaporation dish, evaporated on a water bath, then dried and weighed repeatedly until a constant weight was achieved. The residue mass was obtained by subtracting the mass from the blank test from the constant weight. Finally, the overall migration of the sample was calculated according to the overall migration formula.

The evaluation method for the biodegradability of PTA/PLA/PVA composite bilayer films was conducted with slight modifications to the method described by Xiang et al. (Xiang et al., 2025). Film samples (thickness approximately 44 μm ± 5, area 44.15 cm^2^, weight 0.15 g ± 0.02) were weighed and then buried in a 150 × 300 mm circular glass container filled with soil (plant growing medium) at a pH of 6–7.5, to a depth of approximately 20 cm. During this period, the average daily temperature and relative humidity were 25 °C and 68%, respectively. The samples were excavated at specified time points (0, 5, 15, 30, and 60 days) and photographed for record-keeping. After 60 days, the remaining film fragments were collected, washed, dried, and weighed to calculate the percentage of mass loss.

### 2.4 Monitoring the freshness of pork mince

#### 2.4.1 Pork mince packaging

Fresh pork mince was purchased from a local market (Wuhan, China). Ten grams of fresh pork mince was weighed and placed into a sterilized polyethylene (PE) zip lock bag, with a film (4 cm × 4 cm) attached to the inner side of the polyethylene film. The package was then stored at 4 °C and RH 60% for 4 days, and photographed daily (Wang, Zhao, et al., 2024).

#### 2.4.2 pH, total volatile basic nitrogen (TVB-N) and total viable count (TVC) measurement

First, a 10 g pork mince sample was homogenized with 180 mL of distilled water. The pH of the resulting mixture was then accurately measured using a digital pH meter (PhS-3E, Shanghai INESA Scientific Instrument Co., Ltd., China). Simultaneously, the TVB-N was determined using a semi-micro Kjeldahl nitrogen determination apparatus according to the literature (Choi et al., 2017).

The microbial flora was measured using the plate count method to obtain the TVC. The pork was transferred to a bag containing 135 mL of 0.1 g/dL physiological saline and homogenized for 3 min using a stomacher (JX-05, Tuhe Electromechanical Technology, Shanghai, China). The diluted homogenate was then inoculated onto agar plates and subsequently incubated at 35 °C for 48 h (Yun et al., 2024).

### 2.5 Statistical analysis

The data results are reported as mean ± standard deviation. The release kinetics model data were fitted using Origin software (version 22.0, OriginLab, USA), and one-way analysis of variance was conducted using SPSS software (version 22.0, IBM SPSS Statistics, USA). Duncan's multiple range test was used to evaluate the significant differences between group means, with the significance level set at *p < 0.05*. Each experiment was conducted with at least three parallel sample tests.

## 3 Results and discussion

### 3.1 Mechanical properties

**Fig. 1:**
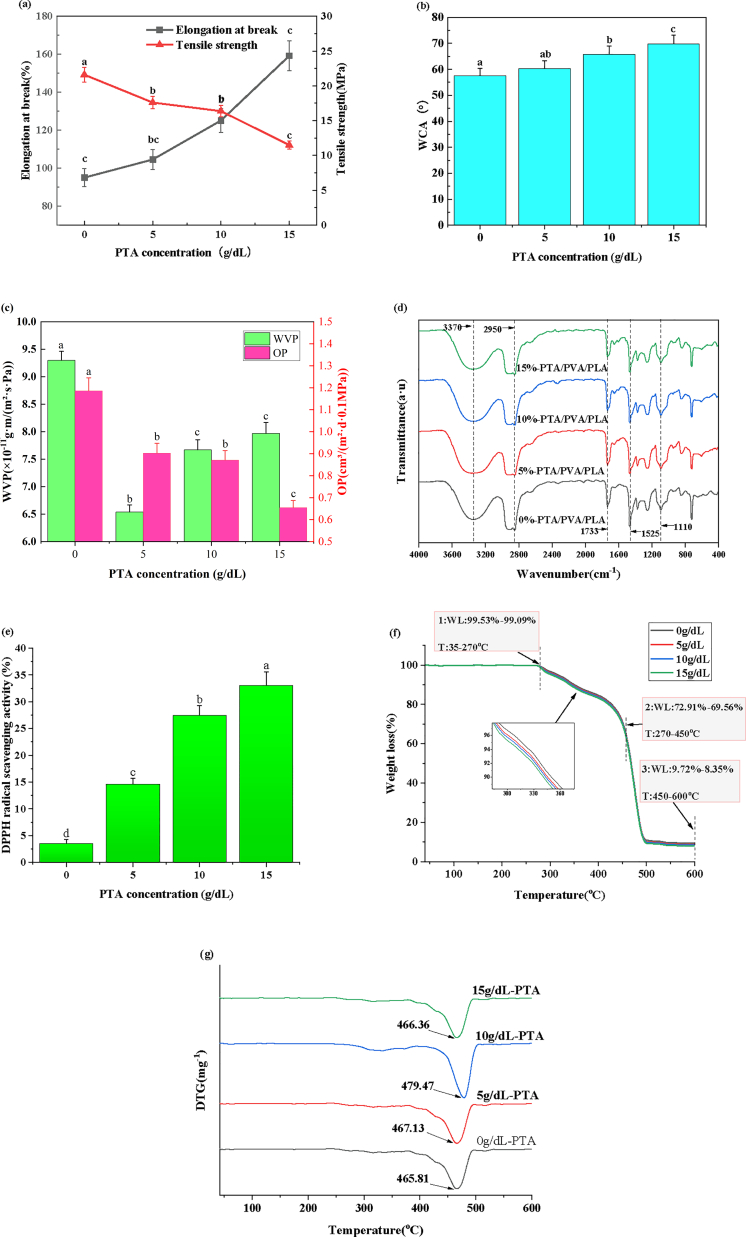
Mechanical properties (a), Water contact angle(b), water vapor transmission rate and Oxygen permeability (c), Fourier transform infrared spectrum (d), DPPH of composite films(e), TGA (f) and DTG (g) of composite films. Different lowercase letters indicate significant differences (*p <* *0.05*).

Our research team's previous study on the mechanical properties of PLA found that the tensile strength and elongation at break were 21.56 MPa and 82.13%, respectively (Yana & Guorui, 2025). As shown in Fig.1a, when the PTA concentration increases from 0 g/dL to 15 g/dL, the elongation at break of the composite film increases with the increase of PTA concentration, while the tensile strength decreases. The tensile strength and elongation at break change from 21.59 MPa and 94.98% to 11.48 MPa and 159.16% (*p < 0.05*), respectively. The results indicate that the elongation at break increases with the increase of PTA concentration, which is attributed to the fact that PTA, as a small molecule substance, can be embedded between polymer chains, that is, into the hydroxyl network of PVA, weakening the hydrogen bonds between chains and acting as a plasticizer. The hydroxyl groups of PTA form a hydrogen bond network with PVA; during the stretching process, the breaking and reformation of hydrogen bonds can absorb energy and delay crack propagation. With the increase of PTA concentration, the tensile strength decreases, which is due to the easy aggregation in the matrix, thus forming defects or stress concentration points. This aggregation leads to the above phenomenon (Mohammadalinejhad et al., 2020).Compared with the tensile strength and elongation at break of PVA monolayer films studied by Jain et al. (Jain et al., 2017) (44 ± 1.5 MPa and 112.5 ± 8.6%, respectively), the results indicate that the mechanical properties of PTA/PVA films based on PLA are higher than those of PVA monolayer films.

### 3.2 Water contact angle

In our previous study, we measured the water contact angle of PLA films before and after plasma treatment; the values before and after treatment were 79.72° and 50.05°, respectively (Yana & Guorui, 2025). As shown in Fig.1b, the water contact angle of the composite film increased from 57.57° to 69.76° (*p < 0.05*) as the PTA concentration rose from 0 to 15 g/dL. This increase is attributable to the hydrophobic aromatic ring structures (e.g., benzene rings) and nonpolar groups present in anthocyanin molecules. At higher PTA concentrations, these molecules may migrate to or self-assemble on the film surface, forming hydrophobic domains that reduce the exposure of hydrophilic hydroxyl (–OH) groups. Concurrently, intramolecular or intermolecular hydrogen bonds may form between PTA and the hydroxyl groups of PVA, which can “lock” the hydrophilic groups of PVA and hinder their full interaction with water molecules, thereby decreasing surface hydrophilicity. In addition, a high PTA concentration may reduce surface roughness by filling microscopic pores within the polymer matrix, resulting in a smoother and denser surface (He et al., 2024). Cai et al. (Cai et al., 2025) found that the incorporation of nonpolar aromatic rings in anthocyanin structures effectively improved the hydrophobicity of nanocellulose composite membranes (with a water contact angle of up to 95°), indicating that anthocyanins can enhance the film hydrophobicity.

### 3.3 Water vapor permeability and oxygen permeability

As shown in Fig.1c, when the PTA concentration increases from 0 g/dL, 5 g/dL, 10 g/dL to 15 g/dL, the water vapor permeability (WVP) decreases from 9.3 × 10^−11^ g·m/(m^2^·s·Pa) to 6.54 × 10^−11^ g·m/(m^2^·s·Pa), and then increases from 7.67 × 10^−11^ g·m/(m^2^·s·Pa) to 7.97 × 10^−11^ g·m/(m^2^·s·Pa) (*p < 0.05*). This indicates that polylactic acid (PLA) films exhibit low water vapor permeability. In this study (Akbari & Mehregan Nikoo, 2025), the WVP of the pristine PLA film was 15.2 × 10^−11^ g·m/(m^2^·s·Pa). However, the incorporation of a PTA/PVA coating effectively enhanced the film's water vapor barrier properties. Sarwar et al. (Sarwar et al., 2018) studied the WVP 43.09 × 10^−11^ g·m/(m^2^·s·Pa) of pure PVA films. The results showed that PTA/PVA films based on PLA exhibited better water vapor barrier properties than single-layer PVA films.

As shown in Fig.1c, as the PTA concentration increased from 0 g/dL to 15 g/dL, the oxygen permeability (OP) of the composite film decreased from 1.18cm^3^/(m^2^·d·0.1 MPa) to 0.65cm^3^/(m^2^·d·0.1 MPa) (*p* *<* *0.05*). Fan et al. (Fan et al., 2025) also observed a similar phenomenon. This is because the hydroxyl groups and other polar groups in PTA molecules may form hydrogen bonds with PVA chains, enhancing intermolecular interactions and thereby increasing the crystallinity or density of the film. The increase in the proportion of crystalline regions reduces the gas diffusion pathways in the amorphous regions, thereby lowering the oxygen permeability. This indicates that the PLA film itself possesses extremely poor barrier properties with an OP of 127.33 cm^3^/(m^2^·d·0.1 MPa) (Aulin et al., 2010), however, when the PLA film is combined with a PTA/PVA coating, its oxygen barrier performance is effectively enhanced. The excellent barrier properties of PTA/PVA-coated films are attributed to the abundant hydroxyl groups (-OH) on the PVA molecular chains. The strong polar interactions (hydrogen bonds) between hydroxyl groups cause the molecular chains to arrange closely, forming highly ordered crystalline regions. Within the crystalline regions, the closely arranged molecular chains reduce diffusion pathways for gas or liquid molecules, thereby enhancing barrier properties. Yeun et al. (Yeun et al., 2006) studied the oxygen permeability of pure PVA films, which was 4.35cm^3^/(m^2^·d·0.1 MPa). The results showed that PTA/PVA films based on PLA exhibited better oxygen barrier properties than single-layer PVA films. And the moisture and oxygen barrier properties were dramatically improved compared with PLA film.

### 3.4 Fourier transform infrared spectroscopy

The Fourier transform infrared (FTIR) spectra of the composite films are shown in Fig.1d. A broad peak was observed at 3370 cm^−1^, attributed to the formation of a hydrogen bonding network between the hydroxyl groups of PTA and PVA. A study by Liu et al. (Liu et al., 2021) also observed a similar phenomenon. This may cause broadening or shifting of the hydroxyl group (O—H) stretching vibration peak. The absorption peak at 2950 cm^−1^ corresponds to the stretching vibration of residual C—O groups in polyvinyl alcohol. Peaks observed at 1733 cm^−1^, 1525 cm^−1^, and 1110 cm^−1^ are deformation vibrations. This may be due to changes in the film's water absorption properties (e.g., increased hydrophobicity due to cross-linking), leading to reduced intensity of the hydroxyl group (O—H) peak, and weakened carbon‑oxygen (C—O) and carbon‑carbon (C—C) vibrations. The FTIR results indicate that the addition of PTA does not alter the chemical structure of PVA/PLA (Marano et al., 2022).

### 3.5 Antioxidant properties

As shown in Fig.1e, the antioxidant activity of the films significantly increased with increasing PTA concentration (*p < 0.05*). The free radical scavenging rates of films containing 0 g/dL, 5 g/dL, 10 g/dL, and 15 g/dL PTA were 3.53%, 14.58%, 27.43%, and 33.06%, respectively. Due to its abundant phenolic hydroxyl groups (particularly the catechol structure) and conjugated system, PTA exerts a potent multi-level antioxidant effect through three primary pathways: direct scavenging of free radicals, chelation of pro-oxidant metal ions, and activation of endogenous antioxidant enzymes (SOD, GSH-Px, CAT). Perron et al. (Perron & Brumaghim, 2009) discovered that chelated metal ions exert antioxidant effects. Zhang et al. (Zhang & Tsao, 2016) discovered that the activation mechanism of enzymes possesses antioxidant effects. This confirms the efficacy and importance of PTA as a natural antioxidant and provides an important scientific basis for the antioxidant design of film materials (Roy & Rhim, 2020).

### 3.6 Thermal stability

The thermal properties of PTA/PVA/PLA films were evaluated using thermogravimetric analysis (TGA) and derivative thermogravimetry (DTG). As seen in Fig.1f, all films exhibited three main stages of mass loss. The first weight loss stage (99.09% to 99.53%) occurred between 30 and 270 °C, primarily due to the evaporation of absorbed water and residual solvents from the films. The second weight loss stage (69.56% to 72.91%) occurred between 270 and 450 °C, attributed to the scission loss of ester bonds in PLA. The third weight loss stage (8.35% to 9.72%) appeared between 450 and 600 °C, caused by the thermal decomposition and carbonization of the film material. The results indicate that PTA content can increase the high-temperature char residue and potential flame retardancy of the films (Zhang et al., 2019).

According to Fig.1g, the main decomposition peaks associated with PTA content are all located within the 460–480 °C interval. The peak temperatures for 0 g/dL-PTA, 5 g/dL-PTA, 10 g/dL-PTA, and 15 g/dL-PTA were 465.81 °C, 467.13 °C, 479.47 °C, and 466.36 °C, respectively. The results show that 10 g/dL-PTA had the highest decomposition peak temperature (479.47 °C), indicating the best thermal stability. This enhanced heat resistance may be due to an appropriate amount of PTA potentially delaying matrix decomposition through cross-linking or flame-retardant mechanisms (Goudarzi et al., 2023).

### 3.7 pH-response

Table 1 shows the color diversity of PTA/PVA/PLA composite films in buffer solutions of different pH values, indicating the excellent pH responsiveness of PTA. When the PTA content was 0 g/dL, the film appeared colorless. With 5 g/dL PTA content, the film displayed a light purplish-red color dominated by PTA under acidic conditions (pH 2–3), light purple at pH 4–5, and gradually faded as the pH increased. Under neutral conditions (pH 7), the film was nearly colorless and transparent. Under alkaline conditions (pH 8–10), the film appeared light yellow, while at pH 11–12, it showed a light green color (Goudarzi et al., 2023).When the PTA content was 15 g/dL, the transparency or gloss of the film was slightly affected. At pH 2 to 5, the film appeared purplish-red; at pH 6 to 7, the color changed to light purple; and at pH 7 to 12, the color gradually changed from light yellow to yellow-green. The results indicate that, through matrix optimization, the PTA/PVA/PLA composite films achieved more precise pH-responsive segmented color-changing capability and may indirectly improve migration issues. A PTA concentration of 10 g/dL achieved the optimal balance in color intensity, transparency, and functionality. Therefore, the composite film with a 10 g/dL concentration holds significant potential as a color-changing material in food packaging (Ma & Wang, 2016).

**Table 1:**
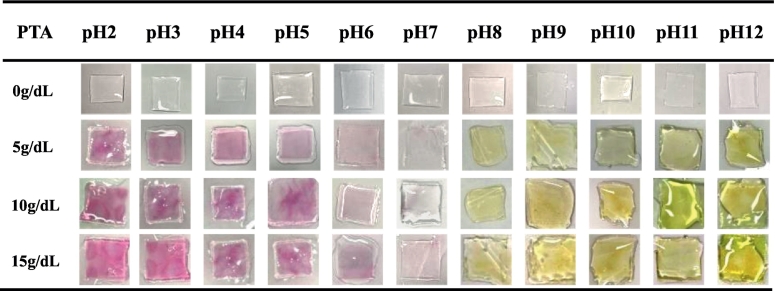
Color reactions of composite films at different pH values.

**Fig. 2:**
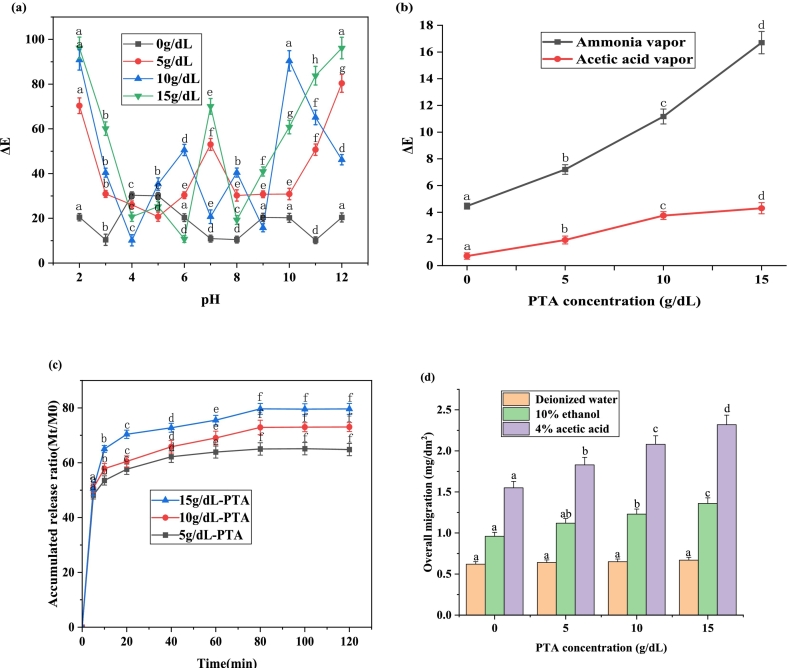
ΔE values of composite films pH(a), ΔE values for the sensitivity of composite films to ammonia and acetic acid(b), Cumulative release curve of PTA from composite films(c),Overall migration of composite films(d). Different lowercase letters indicate significant differences (*p <* *0.05*).

As shown in Fig.2a, different films exhibited distinct variations with pH changes, and the ΔE values were significantly correlated with PTA content. Research shows that ΔE values are closely related to PTA content (*p < 0.05*). Research shows that the changes in ΔE for the 0 g/dL-PTA/PVA/PLA, 5 g/dL-PTA/PVA/PLA, 10 g/dL-PTA/PVA/PLA, and 15 g/dL-PTA/PVA/PLA systems displayed different trends. The pH value exhibited different responses, while the ΔE value depended on the PTA content. The study found that over the pH range 2–12, the ΔE variation values for the 0 g/dL-PTA/PVA/PLA, 5 g/dL-PTA/PVA/PLA, 10 g/dL-PTA/PVA/PLA, and 15 g/dL-PTA/PVA/PLA systems were 0–10, 20–70, 10–90, and 10–96, respectively. This indicates that the 15 g/dL-PTA/PVA/PLA sample exhibited the most significant color change, followed by the 10 g/dL-PTA/PVA/PLA and 5 g/dL-PTA/PVA/PLA samples, while the 0 g/dL-PTA/PVA/PLA sample showed the least noticeable color change. Based on the color change results, PVA films containing 5 g/dL, 10 g/dL, and 15 g/dL PTA possess great potential as color-indicating materials in food packaging (Liu et al., 2022).

### 3.8 Sensitivity to ammonia and acetic acid and color reversibility

Fig.2b presents the color changes of composite films with varying PTA concentrations in ammonia and acetic acid vapor environments (*p < 0.05*). As PTA concentration increases, film sensitivity to ammonia progressively improves, with ΔE exhibiting an overall upward trend, indicating enhanced ammonia detection capability. At PTA concentrations of 0 g/dL, 5 g/dL, 10 g/dL, and 15 g/dL, ΔE values reach 4.46, 7.20, 11.17, and 16.70, respectively. This enhancement is attributed to increased active sites for ammonia interaction at higher PTA concentrations, yielding more pronounced color changes. These results demonstrate that elevated PTA concentrations significantly enhance ammonia sensitivity. PTA concentration variation minimally affects acetic acid vapor sensitivity. Corresponding ΔE values measure 0.72, 1.92, 3.75, and 4.30 at 0 g/dL, 5 g/dL, 10 g/dL, and 15 g/dL PTA, with limited ΔE magnitude variation. This reduced sensitivity likely stems from acetic acid vapor's distinct chemical properties compared to ammonia (Zhu et al., 2021). The films exhibit robust color-responsive characteristics for both analytes, with higher PTA concentrations amplifying response magnitude. Ammonia exposure distinctly intensifies yellowish-green hues, while acetic acid vapor enhances pink coloration.

In the field of food packaging, reversible color changes can provide crucial information regarding food freshness and storage conditions. To evaluate film reversibility, this study conducted tests by repeatedly exposing the films to concentrated ammonia solution (alkaline) and acetic acid solution (acidic). As shown in Table 2, the films exhibited rapid color changes in both acidic and alkaline environments: transitioning to yellowish-green under alkaline conditions and pinkish-purple under acidic conditions. However, repeated pH cycling may impose certain effects on both the structural integrity of the film and the stability of anthocyanins. Anthocyanins undergo reversible structural transformations among different ionic forms (flavylium cation, quinoidal base, hemiketal, and chalcone) depending on the pH environment. While these transformations are inherently reversible, repeated cycling between acidic and alkaline conditions may promote irreversible degradation pathways, particularly under strong alkaline conditions where anthocyanins are prone to degradation. This degradation is manifested as gradual color darkening and decreased uniformity with increasing cycle numbers (Zhai et al., 2017).

**Table 2:**
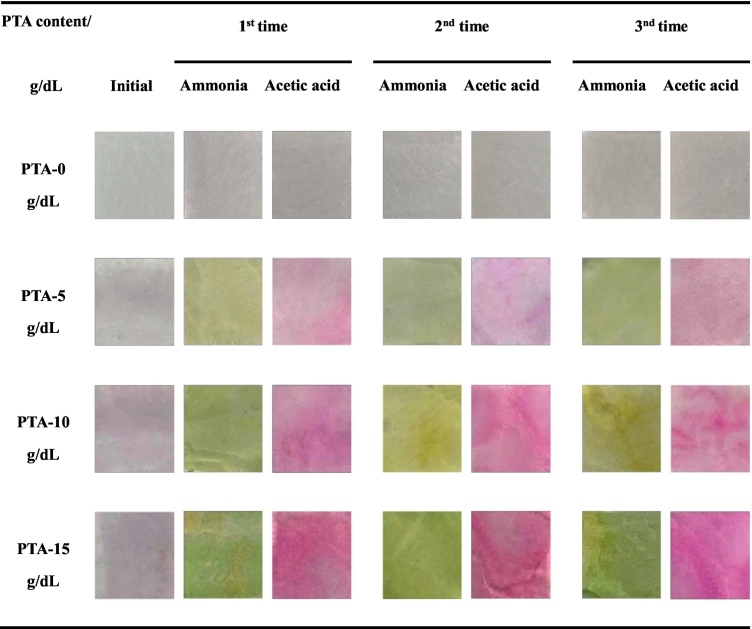
Reversible color change of the films.

Furthermore, higher PTA content resulted in more pronounced color changes. When transitioning from ambient air to alkaline solution, the film color changed instantaneously from light purple to yellowish-green within 1 s. After removal from the alkaline environment, the color rapidly changed from yellowish-green to purple within 5 s. Similarly, rapid color changes occurred when exposed to acidic solution, transitioning from purple to pink (transition time < 30 s). The films' high pH sensitivity originates from their porous structure and stable hydrophilic/hydrophobic balance, facilitating uniform vapor diffusion and rapid release (Lin et al., 2022).

### 3.9 Release behavior of PTA in films

As shown in Fig.2c, the cumulative release rate of anthocyanins in a 95 g/dL ethanol solution exhibits an initial rapid increase followed by a gradual stabilization over time (*p < 0.05*). The highest release rate was observed under 15 g/dL PTA conditions, reaching nearly 80% after 120 min. This is attributed to the synergistic effect of high temperature, enhancing diffusion kinetics and solubility equilibrium. Under 10 g/dL PTA conditions, the release rate was lower, while under 5 g/dL PTA conditions, it was the lowest at approximately 50%. This is due to slow molecular diffusion at low temperatures and low solubility, resulting in unreleased anthocyanins remaining in the carrier. This phenomenon indicates that higher temperature conditions significantly enhance anthocyanin release efficiency. As time increases, especially after 70 min, the release differences between concentrations become significant (Li et al., 2013).

### 3.10 Overall migration tests

As shown in Fig.2d, the overall migration of the film in the aqueous simulant ranged from 0.62 mg/dm^2^ to 0.67 mg/dm^2^ as the PTA concentration increased from 0 g/dL to 15 g/dL, indicating limited release of PTA (*p < 0.05*). Overall migration in this medium primarily depended on the hydrophilicity of PVA and the water-soluble components within the film, resulting in the lowest overall migration levels. In the 10% ethanol simulant, overall migration increased from 0.96 mg/dm^2^ to 1.36 mg/dm^2^ with PTA concentrations from 0 g/dL to 15 g/dL. Ethanol, as an organic solvent, exhibits affinity for both PLA (hydrophobic) and PVA (hydrophilic). It may slightly plasticize the polymer matrix, enhance segment mobility, and improve the solubility of hydrophobic anthocyanins in the simulant, thereby promoting overall migration. In the 4% acetic acid simulant, overall migration levels ranged from 1.55 mg/dm^2^ to 2.31 mg/dm^2^ across the same PTA concentration range. Acetic acid may induce partial hydrolysis or swelling of ester or ether bonds in the PLA/PVA matrix, increasing the porosity of the polymer network. This reduces diffusion resistance, facilitating the overall migration of anthocyanins and small-molecule matrix components. The maximum overall migration value observed was only 2.32 mg/dm^2^, which is well below the internationally accepted total overall migration limit of 10 mg/dm^2^.

Regarding the toxicological implications of the released substances, the safety of PVA and anthocyanins in food-related applications has been extensively evaluated. Polyvinyl alcohol (PVA) is authorized as a food additive in the European Union in accordance with Annex II to Regulation (EC) No 1333/2008, and its safety has been critically evaluated by multiple regulatory authorities (EFSA Panel on Food Additives and Nutrient Sources added to Food (ANS), 2013). Anthocyanins are naturally occurring pigments with a long history of dietary exposure. The elevated anthocyanin levels in purple tomatoes do not pose a hazard to health, and their content does not reach levels that would raise safety concerns.

This indicates excellent overall chemical stability of the film substrate, with very low total leachates under the conditions tested, meeting basic safety requirements. This finding is consistent with the increased overall migration trends reported for PLA films in previous studies, such as that by Redfearn et al. (Redfearn & Goddard, 2023).

### 3.11 Biodegradation analysis

**Fig. 3:**
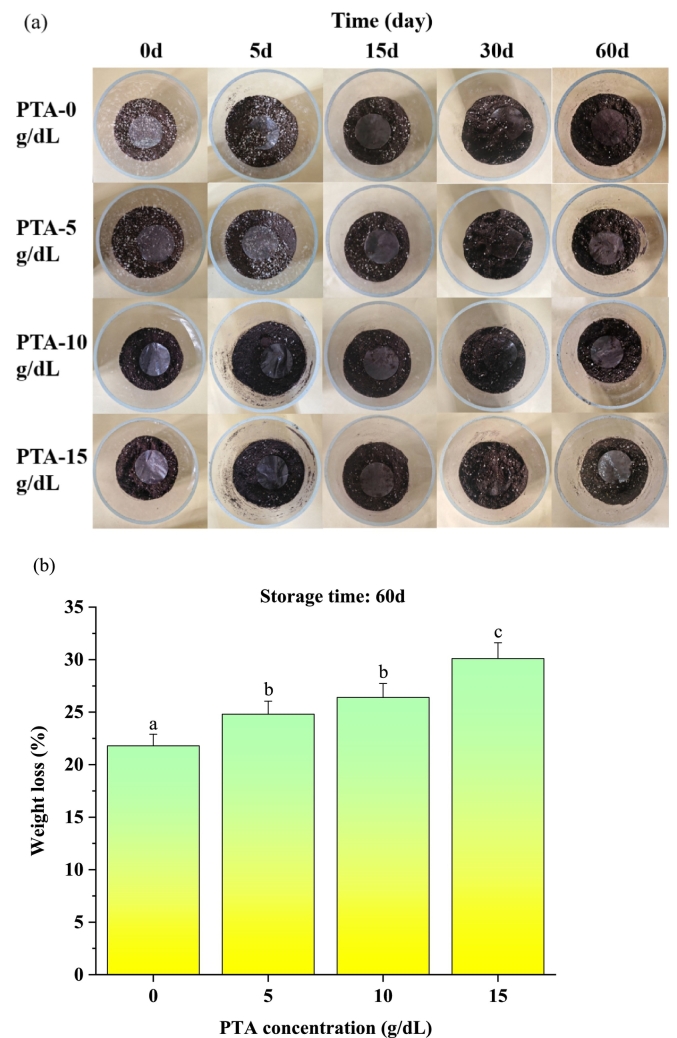
Duration of soil burial for composite films (a) and biodegradation rate of composite films (b).

In practical applications, the biodegradability of materials has become a key indicator of their environmental performance (Luan et al., 2024). In this study, the biodegradability of various PTA/PVA/PLA composites was investigated through soil burial tests, and the visual changes and mass loss over different degradation periods were analyzed. Fig.3a shows that the appearance of films with different concentrations did not undergo significant changes after soil burial. Fig.3b shows that the mass loss for anthocyanin concentrations of 0 g/dL, 5 g/dL, 10 g/dL, and 15 g/dL was 21.8%, 24.8%, 26.4%, and 30.1%, respectively (*p < 0.05*). The results indicate that as the anthocyanin concentration increases, the film's degradability also increases accordingly. This may be attributed to the carboxyl groups in PTA molecules forming hydrogen bonds with the hydroxyl groups of PVA, disrupting the original hydrogen bond network between PVA molecular chains. Simultaneously, this disrupts the crystalline regions of PLA, allowing water molecules or degradation agents to penetrate the film more easily, thereby accelerating hydrolysis or enzymatic degradation processes. Meng et al. (Meng et al., 2025) found that pure PLA films degraded to 0.13% after 60 days of natural degradation. The addition of PVA not only accelerated the degradation of the film but also significantly enhanced the degradation of PLA, consistent with observations reported in the literature.

### 3.12 Morphology

**Fig. 4:**
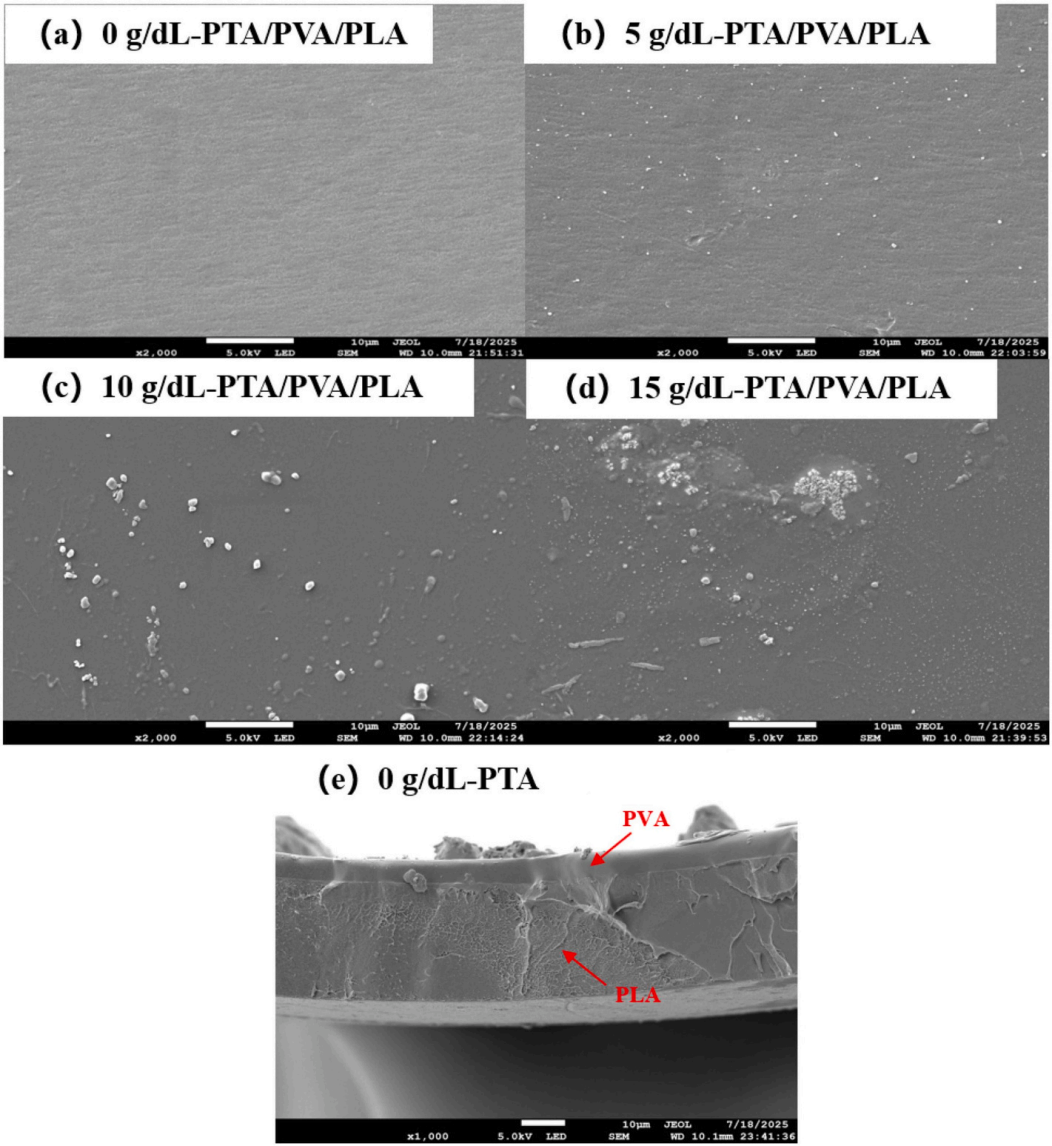
SEM images of film, surface: (a), (b), (c), (d); fracture surface: (e).

The SEM images of PTA/PVA/PLA films are shown in Fig. 4. As shown in Fig.4a, 0 g/dL-PLA has a homogeneous surface. However, after adding PTA, as shown in Fig.4b, particle-like substances appeared on the surface of the 5 g/dL-PTA/PVA/PLA film. As shown in Fig.4c, the surface of the 10 g/dL-PTA/PVA/PLA film had particles similar to grains. As shown in Fig.4d, the surface of the 15 g/dL-PTA/PVA/PLA film had aggregated particles. In summary, as the anthocyanin content increased, the surface particles became larger, which might be due to the aggregation of the purple tomato anthocyanins. They would become rough and present a granular structure (Liu et al., 2024). As shown in Fig.4e, from the cross-sectional view, it can be seen that the film of 0 g/dL-PLA was made of two layers, with that the upper layer being PVA coating with about 5 μm, and the lower layer being PLA as the substrate with about 30 μm. The cross-section presents wrinkled, stretched fibers and fine cracks, being characterized by ductile fracture, which was consistent with the properties of PLA (Yun et al., 2021).

### 3.13 Monitoring the freshness of pork mince

Proteins in meat products are highly susceptible to spoilage caused by bacterial and fungal activity. During this process, various nitrogen-containing volatile compounds are produced through decomposition. These alkaline nitrogen-containing compounds alter the molecular structure of the indicator PTA in the film, resulting in corresponding changes in the film's color. As shown in Table 3, the pH value of pork mince increases with extended time, and changes in pH value are correlated with the freshness of the pork mince. The results indicate that on Day 0, a pH value of 5.41 indicates fresh pork mince; on Day 1, a pH value of 5.48 indicates fresh pork mince; on day 2, a pH value of 5.63 indicates fresh pork mince; on day 3, a pH value of 6.17 indicates slightly less fresh pork mince; on day 4, a pH value of 6.65 indicates spoiled pork mince (Cao et al., 2023).

**Table 3:** Changes in freshness (TVC and pH value) and film AE value of pork mince stored at 25 °C, and changes in pork freshness color monitored through the film.

Time	TVC	pH	TVB-N	∆E			
(d)	(log CFU/g)		(mg/100 g)	PTA-0 g/dL	PTA-5 g/dL	PTA-10 g/dL	PTA-15 g/dL
0	4.42 ± 0.04^c^	5.41 ± 0.01^d^	8.17 ± 0.15^e^	–	–	–	–
1	4.86 ± 0.06^d^	5.48 ± 0.02^d^	10.85 ± 0.24^d^	0.41 ± 0.39^a^	3.74 ± 1.14^a^	7.48 ± 1.52^d^	8.26 ± 2.03^d^
2	5.44 ± 0.05^c^	5.63 ± 0.02^c^	13.17 ± 0.31^c^	0.64 ± 0.56^c^	1.61 ± 0.69^c^	1.91 ± 1.77^c^	7.57 ± 1.31^c^
3	5.92 ± 0.02^b^	6.17 ± 0.01^b^	14.87 ± 0.17^b^	0.71 ± 0.77^b^	2.37 ± 0.81^b^	5.06 ± 1.23^b^	3.54 ± 1.62^b^
4	6.13 ± 0.03^a^	6.65 ± 0.01^a^	17.96 ± 0.22^a^	0.39 ± 0.36^a^	3.86 ± 1.46^a^	9.54 ± 0.51^a^	5.71 ± 2.15^a^

Note: Data in the table are expressed as mean ± standard deviation; different lowercase letters indicate statistically significant.

TVB-N is a key chemical indicator for assessing the decline in freshness and degree of spoilage of pork mince and other meats, with its content significantly increasing due to protein degradation caused by microorganisms. Research indicates that the TVB-N content in fresh pork mince is below 15 mg per 100 g. As shown in Table 3, TVB-N levels increased from 8.17 mg/100 g on Day 0 to 10.85 mg/100 g on Day 1, 13.17 mg/100 g on Day 2, 14.87 mg/100 g on Day 3, and 17.96 mg/100 g on Day 4. The results indicate that by Day 4, the pork mince had already spoiled (Sikora et al., 2023).

Microbial growth is the primary cause of spoilage in pork mince. The total colony count during storage reflects the microbial growth on the pork mince. When the total colony count exceeds 6.01 log CFU/g, the pork mince is considered spoiled. As shown in Table 3, the TVC value on Day 0 was 4.42 log CFU/g, on Day 1 it was 4.86 log CFU/g, on Day 2 it was 5.44 log CFU/g, on Day 3 it was 5.92 log CFU/g, and on Day 4 it was 6.13 log CFU/g. By Day 4, the total colony count had exceeded the upper limit of the safety standard, indicating that the pork mince had spoiled (Atarés & Chiralt, 2016).

As shown in Table 4, the color of the PTA/PVA/PLA composite film used for pork mince packaging changes with increasing PTA content. When the PTA content is 0 g/dL, the film does not undergo any color changes during storage. In a 5 g/dL PTA film, the color changes from light purple on day 0 to day 2, indicating fresh pork mince; on day 3, the color fades, indicating slightly less fresh pork mince; on day 4, the color changes to light green, indicating spoiled pork mince. In a 10 g/dL PTA film, the color changes from light purple on day 0 to day 2, indicating fresh pork mince; on day 3, it turns light yellow-green, indicating that the pork mince is less fresh; on day 4, it turns yellow-green, indicating that the pork mince has spoiled. As shown in Table 3, the ΔE value for 10 g/dL PTA film on Day 4 is 9.54 ± 0.51, indicating significant color difference changes. For the 15 g/dL PTA film, the color on days 0 and 1 was light purple, indicating that the minced pork was fresh; on days 2 and 3, it turned to light purplish-green, indicating that the minced pork was less fresh; on day 4, it turned to yellow-green, indicating that the minced pork had spoiled.

**Table 4:**
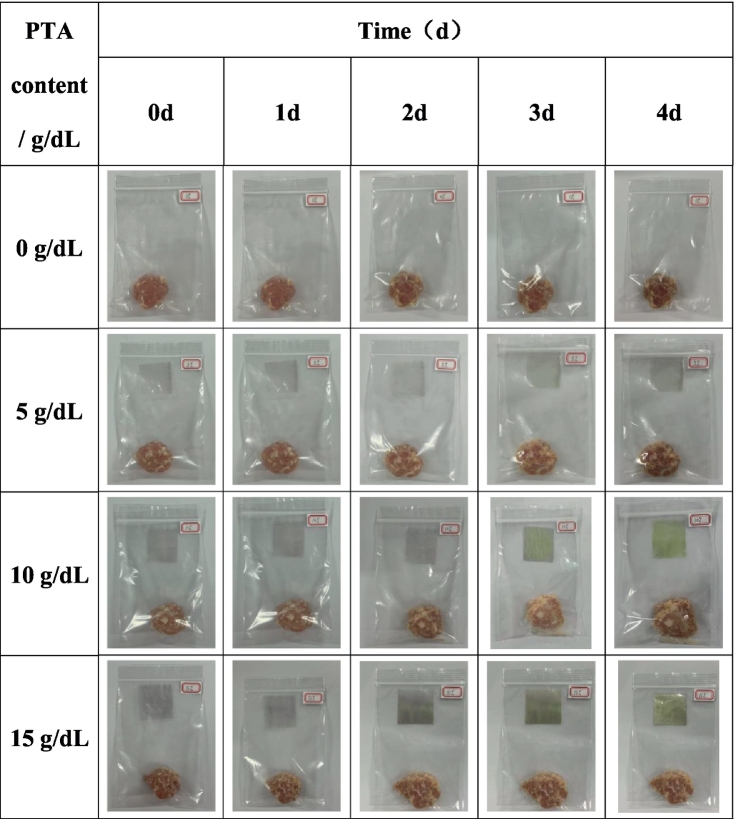
Color changes in PTA/PVA/PLA films used to monitor the freshness of pork mince stored at 25 °C.

Thus, during the storage of pork mince, the noticeable color changes on the film indicate that different films can be selected for different purposes. Suppose indicating freshness or spoilage, 15 g/dL PTA can be chosen. If indicating completely fresh or slightly fresh, then 10 g/dL PTA should be selected, providing the best color response and safety in pork mince packaging (Atarés & Chiralt, 2016).

## 4 Conclusions

In this study, biodegradable polylactic acid (PLA) film was used as the substrate matrix. Plasma treatment modified the PLA film surface, enhancing its hydrophilicity and thereby improving its affinity with the PTA/PVA coating. A PTA/PVA solution served as the coating liquid and was applied onto the PLA surface to prepare PLA-based PVA/anthocyanin composite films with anthocyanin contents of 0 g/dL, 5 g/dL, 10 g/dL, and 15 g/dL, respectively. The results indicate that anthocyanin concentration had no significant effect on the thickness of the composite films. With increasing anthocyanin concentration, the elongation at break of the composite films gradually increased while the tensile strength gradually decreased, indicating that PTA incorporation effectively enhanced the toughness of the composite films but reduced their rigidity. Concurrently, the water contact angle of the composite films increased with rising anthocyanin concentration, indicating reduced hydrophilicity of the composite films. This suggests that high anthocyanin concentrations may fill microscopic pores in the polymer matrix, reduce surface roughness, and form a smoother, denser surface. Oxygen transmission rate testing indicated that the oxygen transmission rate of the composite film decreased with increasing anthocyanin concentration. This signifies that the film achieved optimal oxygen barrier performance at an anthocyanin concentration of 15 g/dL. Overall migration tests in food simulants demonstrate that the film's maximum overall migration value of 2.31 mg/dm^2^ is substantially below the European Union overall migration limit for food-contact polymers (10 mg/dm^2^). Biodegradation tests indicate that the degradation rate of the film increases with increasing PTA content. The pH responsiveness and pork packaging experiments demonstrated that the composite film containing 10 g/dL PTA exhibited significant color changes, indicating its excellent responsiveness in monitoring the freshness of ground pork.

In summary, the optimal PTA content was 10 g/dL, the PTA/PVA/PLA composite films with a PTA content of 10 g/dL exhibited relatively outstanding mechanical properties, with tensile strength and elongation at break of 164.36 MPa and 125.04%, respectively. The indicator functionality changes from light purple to yellow-green, indicating that the pork mince has spoiled. The water contact angle 65.73°, barrier performance oxygen permeability and water vapor permeability are 0.87 cm^3^/(m^2^·d·0.1 MPa) and 7.67 × 10^−11^ g·m/(m^2^·s·Pa), respectively, and thermal stability decomposition temperature 479.47 °C. But our research has found that as the anthocyanin (PTA) concentration increases, the elongation at break (toughness) of the film improves, while the tensile strength (rigidity) decreases significantly. This implies that films with high PTA content may become softer and more deformable, making them more susceptible to tearing or puncturing during packaging transportation and handling, thereby compromising their mechanical protective function.

However, compared to monolayer films of pure PLA or PVA films, the prepared bilayer composite film offered superior mechanical and barrier properties. This PTA/PVA-based PLA biodegradable smart indicator film addresses the dual demands of the food packaging industry for sustainability and food waste reduction, providing an eco-friendly smart packaging solution that enhances food safety while minimizing economic losses. Meanwhile, the pronounced pH responsiveness of anthocyanins provides real-time visual freshness monitoring for consumers and retailers. The biodegradability (polylactic acid substrate), smart indicator functionality (anthocyanin), and high barrier performance (composite structure) position this film for broad application prospects in the future field of smart food packaging.

## CRediT authorship contribution statement

**Guorui Zhou:** Writing – review & editing, Writing – original draft, Visualization, Validation, Formal analysis. **Yana Li:** Writing – review & editing, Validation, Supervision, Methodology, Data curation. **Beihai Wang:** Resources, Project administration, Investigation, Funding acquisition.

## Funding

This study was not funded by any financial support.

## Declaration of competing interest

The authors declare that they have no known competing financial interests or personal relationships that could have appeared to influence the work reported in this paper.

## Data Availability

Data will be made available on request.
