# Corrigendum to “Plasma-treated PLA incorporated with purple tomato anthocyanins-based coating to fabricate bilayer indicator films for monitoring the freshness of minced pork” [Food Chem.: X 38 (2026) 104247]

**DOI:** 10.1016/j.fochx.2026.104366

**Published:** 2026-08-29

**Authors:** Guorui Zhou, Yana Li, Beihai Wang

**Affiliations:** School of Mechanical Engineering, Wuhan Polytechnic University, Wuhan 430023, China

The authors regret that in the original publication of this article, an error was introduced in the “Results and Discussion” section, subsection 2.4.1 “Pork mince packaging”. The storage temperature of the pork mince samples was incorrectly stated as 4 °C. The correct temperature should be 25 °C, which is consistent with the experimental design described in the “Materials and Methods” section. The authors confirm that this correction does not affect the scientific conclusions of the study.

This research was supported by the Emerging Interdisciplinary Research Funding of WHPU (NO.WHPU-EIRF-2616).

The authors would like to apologise for any inconvenience caused.

